# Frequent Mental Distress, Chronic Conditions, and Adverse Health Behaviors in the Behavioral Risk Factor Surveillance Survey, Jordan, 2007

**DOI:** 10.5888/pcd10.130030

**Published:** 2013-08-22

**Authors:** Mohannad Al-Nsour, Meyasser Zindah, Adel Belbeisi, Italia V. Rolle, Henry Walke, Tara Strine, Geraldine S. Perry, Bassam Jarrar, Ali Mokdad

**Affiliations:** Author Affiliations: Meyasser Zindah, Adel Belbeisi, Jordan Ministry of Health, Amman, Jordan; Italia V. Rolle, Henry Walke, Tara Strine, Geraldine S. Perry, Bassam Jarrar, Centers for Disease Control and Prevention, Atlanta, Georgia; Ali Mokdad, University of Washington, Seattle, Washington.

## Abstract

**Introduction:**

Recent evidence indicates that chronic diseases and mental illness are associated. In the Middle Eastern country of Jordan, chronic diseases and frequent mental distress (FMD) are increasing; however, the capacity for mental health care is limited. The objective of this study was to determine the association between FMD, chronic conditions, and adverse health behaviors in Jordan.

**Methods:**

The third cycle of the Jordan Behavioral Risk Factor Surveillance Survey (2007) served as the data source for this study. The sample consisted of 3,612 noninstitutionalized Jordanian adults aged 18 years or older. Logistic regression was used to obtain odds ratios for the association between chronic conditions, health behaviors, and FMD adjusted for age, sex, marital status, education, income, and employment.

**Results:**

In the adjusted models, people with hypertension (adjusted odds ratio [AOR], 2.0; 95% confidence interval [CI], 1.6–2.7), high cholesterol (AOR, 2.3; 95% CI, 1.6–3.2), diabetes (AOR, 1.6; 95% CI, 1.1–2.4), and asthma (AOR, 2.2; 95% CI, 1.5–3.1) and smokers (AOR, 1.5; 95% CI, 1.1–2.0) were more likely to have FMD than people without each of these conditions. Adults who reported vigorous physical activity were less likely to have FMD (AOR, 0.6; 95% CI, 0.4–0.9) than their less active counterparts.

**Conclusions:**

In Jordan, FMD was associated with several chronic conditions. As a result, we suggest additional research to examine the complex relationship between FMD and chronic conditions. More doctors in the primary health care system should be trained in mental health.

## Introduction

Heart disease and depression are predicted to be the top 2 contributors to the global burden of disease by 2020 (1). Evidence from the literature links mental health and chronic diseases, suggesting the need to further examine this association. In a review article by Chapman et al (2), an association was noted between depression and major chronic diseases such as asthma, arthritis, cardiovascular disease, cancer, diabetes, and obesity. These researchers suggested that depression is a key factor in chronic disease occurrence and outcome. A study of disability, mortality, and interactions between chronic diseases and mental illness also reported associations between depression and cardiovascular disease, hypertension, and diabetes (3). Similarly, data from the World Health Organization (WHO) health survey examining depression and chronic diseases at a global level found that depression co-existed with other chronic diseases such as diabetes, arthritis, and asthma (4). The well-established body of mental health and chronic disease research work that exists is focused in high-income countries. However, in low- and middle-income countries, where historically research has focused primarily on infectious diseases (5), there is limited evidence regarding the relationship between mental illness and chronic diseases.

Many low- and middle-income countries are experiencing an epidemiologic transition in which infectious diseases are declining and chronic diseases are increasing, attributed in part to urbanization, globalization, and the adoption of westernized lifestyles. The Middle Eastern country of Jordan is experiencing this transition. Studies in Jordan have investigated chronic diseases, including cardiovascular disease and related risk factors (6–12). Findings from the 2002, 2004, and 2007 population-based Jordan Behavioral Risk Factor Surveillance Survey (BRFSS), which was modeled after the US Centers for Disease Control and Prevention’s BRFSS, indicated that high blood pressure, high blood cholesterol, diabetes, heart disease, and asthma were major chronic diseases affecting the adult population (13,14). Obesity, lack of physical activity, and cigarette smoking were also prevalent. A comparison of the 2002 and 2004 Jordan BRFSS to the 2007 survey showed the prevalence of diabetes, heart disease, and asthma were increasing in Jordan (14). In 2004, the prevalence of frequent mental distress (FMD) among adults aged 18 years or older in Jordan was 5.7% (15); by 2007, the prevalence was 10.3% (14). Before 2004 there was little data on the prevalence of mental illness in Jordan. A review of mental health-related studies from the Middle East revealed few studies addressing mental health and illness (16–20) compared with chronic diseases, perhaps because of the stigma associated with mental illness in this area. Further, the focus of the mental health-related studies was mainly related to use of mental health services (17,18), risk factors for depression (19), and barriers to mental health treatment (16). No studies examined the relationship between mental health and chronic disease.

An assessment of Jordan’s mental health system by WHO revealed the lack of an official mental health policy, dedicated funds in the government budget for mental health care, and limited training of doctors and nurses in the primary health care system on mental illness (21). With regard to the number of mental health facilities, WHO reports 2 mental health hospitals with 330 beds, 64 outpatient facilities, 1 day treatment facility, and 1 community residential facility with 150 beds (21). Most in-hospital mental health services are in the capital area of Jordan/Amman (22). An estimated 1.2 psychiatrists per 100,000 persons serve the mental health population in Jordan; an additional 6.9 psychologists support mental health care in the country (22). A survey of Jordanian primary health care physicians (n = 115) indicated that very few (4.4%) felt competent treating mental health patients; however, 83% of the physicians were supportive of receiving mental health training (22). Less than 40% of physicians thought mental health patients should be treated in the primary health care system (22).

The proportion of people thought to be affected by mental illness in Jordan is approximately 15.7% (21). Obtaining the prevalence of mental illness in Jordan is not easy at this stage because of limited data. An alternative to examining mental illness is FMD, defined as ≥14 mentally unhealthy days in the previous 30 days. FMD is used as a proxy for mental illness (23). Previous investigations indicate that this definition is comparable to the number of days associated with clinical depression (23–26). Because of the rise in the prevalence of chronic disease and the apparent relationship between chronic disease and mental illness, assessing this relationship in the context of Jordan may help inform mental health practices. In our study, we use data from the 2007 Jordan BRFSS to 1) examine the prevalence of FMD by sociodemographic factors and 2) investigate the association between FMD and selected chronic conditions and adverse health behaviors.

## Methods

In 2007, the Jordanian Ministry of Health administered the third cycle of the BRFSS. The household survey is a nationally representative sample of noninstitutionalized adults aged 18 years or older and is designed to gather self-reported information on demographic factors, health status, chronic diseases, and health behaviors. The questionnaire was pilot tested in Jordan. A multistage sampling design was used. One adult aged 18 years or older in each household was selected at random and interviewed in person in the home. The sampling design for the 2007 Jordan BRFSS was the same as for the 2004 survey (15). The survey was conducted in the field between June 1 and August 23, 2007, and had a response rate of 99.1%. There were 3,654 participants; 42 were excluded because of missing information on mentally unhealthy days, for a final analytic sample of 3,612. The BRFSS was approved by Jordan’s Ministry of Health ethical review board.

For the purposes of this analysis, variables were divided into 4 categories: sociodemographics, chronic conditions, health behaviors, and mental health status. For demographics, age was divided into 4 categories (18–34, 35–49, 50–64, and 65 years or older); current marital status consisted of 2 categories (married and not married [single, divorced, widowed, separated]); education was divided into none/primary, secondary/technical school, and college; and monthly household income was reported in Jordanian dinars and had 3 categories reflecting low, moderate, and high income (<100, 100–299; and ≥300 dinars, respectively), which are equivalent to <US$140, US$140–$419, and >US$419, respectively. Employment was categorized as employed (government employee, nongovernment employee, self-employed) and unemployed (volunteer, homemaker, student, retired, unemployed).

In the 2007 BRFSS, the chronic conditions assessed were hypertension, heart disease, heart failure, rheumatic fever, heart attack, asthma, high blood cholesterol, and diabetes. Health behaviors assessed were moderate and vigorous physical activity, tobacco use, diet, and preventive screenings. For the purposes of this study, we chose to focus only on asthma, diabetes, hypertension, high blood cholesterol, vigorous physical activity, and smoking. Our selection of these conditions was based on the quality of the data including missing data and clarity of the question asked to survey participants. Self-reported chronic conditions were assessed by asking, “Have you ever been told by a health professional that you have this condition?” High cholesterol was assessed by asking, “Have you ever been told by a health professional that your blood cholesterol is high?” Chronic conditions (hypertension, high cholesterol, diabetes excluding gestational diabetes, and asthma) were coded as yes and no.

Smoking was determined from the question “Do you currently smoke any tobacco products such as cigarettes, cigars, or pipes?” Smoking was coded as yes if the participant was a current tobacco smoker. Vigorous activity was assessed by asking, “Do you do any vigorous-intensity sports, fitness or recreational (leisure) activities that cause large increases in breathing or heart rate (like running or football) for at least 10 minutes continuously?” Vigorous activity was categorized as yes if the participant reported 10 minutes or more of leisure vigorous activity. Number of days and minutes of vigorous activity were also asked; however, 88% of the responses were missing.

Mental health status was assessed by asking: “Now thinking about your mental health, which includes stress, depression, and problems with emotions, for how many days during the past 30 days was your mental health not good?” FMD was defined as ≥14 mentally unhealthy days and used as a proxy for poor mental health status. FMD was the outcome variable.

Univariate, bivariate, and multivariable analyses were conducted by using the appropriate survey commands in Stata SE version 11 (StataCorp LP, College Station, Texas) to account for the complex sampling design of the BRFSS. Logistic regression was used to obtain odds ratios (ORs) for the association of each chronic condition and health behavior and FMD adjusted first by age and sex, and second by age, sex, marital status, education, employment, and income. The analyses were conducted separately for each chronic condition and health behavior. Age, sex, education, income, and employment were treated as potential confounders in the analysis. A relative standard error of less than 30% was used to determine whether the estimates were stable. Adjusted Wald tests were used to examine whether FMD varied significantly among subgroups for demographic factors and chronic conditions. Significance was set at *P* < .05.

## Results

The overall prevalence of FMD was 10.3%. Adults aged 18–34 years had a significantly lower prevalence of FMD (8.2%) compared with adults 35–49 years (10.2%), 50–64 years (13.4%) and those 65 years or older (11.9%) (Table 1). Women had a significantly higher prevalence of FMD (11.8%) than men (9.0%). There were no significant differences noted by marital status or educational level. Jordanian adults with a low monthly household income had a significantly increased prevalence of FMD (18.4%) than those with higher incomes. The prevalence of FMD was also significantly higher among unemployed Jordanian adults than those adults who were employed.

**Table 1:** Prevalence of Frequent Mental Distress^a^ by Sociodemographic Characteristics Among Adults Aged 18 Years or Older, Behavioral Risk Factor Surveillance Survey (BRFSS), Jordan, 2007

Characteristic	Sample Size^b^	Frequent Mental Distress, % (95% CI)^c^	*P* Value^d^
**Total**	3,612	10.3 (9.3–11.3)	NA
**Age, y**			
18–34	1,245	8.2 (6.4–10.0)	.023^e^
35–49	1,324	10.2 (8.5–11.8)	
50–64	671	13.4 (10.7–16.0)	
≥65	372	11.9 (8.4–15.4)	
**Sex**			
Male	1,932	9.0 (7.7–10.4)	.004^e^
Female	1,680	11.8 (10.4–13.2)	
**Marital status**			
Married	2,632	10.4 (9.1–11.6)	.822
Not married	980	10.1 (8.1–12.1)	
**Education**			
None/primary	1,594	11.6 (9.8–13.4)	.108
Secondary/tech	1,520	9.7 (8.3–11.2)	
College	498	7.8 (5.0–10.6)	
**Monthly income**			
Low (<US$140)	326	18.4 (13.5–23.2)	.001^e^
Medium (US$140–$419)	2,245	9.5 (8.4–10.7)	
High (>US$419)	936	8.8 (6.9–10.8)	
**Employment**			
Employed	1,456	8.0 (6.4–9.5)	.0001^e^
Unemployed	2,154	11.9 (10.5–13.2)	

Abbreviation: CI, confidence interval; NA, not applicable.

a Defined as 14 or more mentally unhealthy days in the past 30 days.

b Numbers may not equal total sample because of missing data.

c Estimates and confidence intervals take into account the complex survey design of the Jordan BRFSS.

d Adjusted Wald test for differences.

e Significance at *P* < .05.

The prevalence of FMD was approximately twice as high among people with hypertension (17.3%) compared with those without hypertension (8.8%). The difference was similar between people with high cholesterol (19.8%) compared with those with normal cholesterol (9.4%) and between people with asthma (20.2%) compared with those without asthma (9.6%) (Table 2). People with diabetes also had a higher prevalence of FMD (17.2%) than those without (9.6%). We found significant differences by adjusted Wald tests. The difference in FMD prevalence between smokers and nonsmokers (11.1% vs 10.0%) was not significant. Adult Jordanians who reported vigorous physical activity had a lower prevalence of FMD than those who did not report vigorous physical activity (6.2% and 10.9%, respectively) (Table 2). In the unadjusted models, people with hypertension, high cholesterol, diabetes, and asthma were significantly more likely to have FMD than those without each of these conditions (Table 2). There was a 10% increase of FMD among tobacco smokers compared with nonsmokers; however, in the unadjusted model the association was not significant. After adjusting for age and sex, adults with the following chronic conditions were significantly more likely to report FMD than those without: hypertension (adjusted ratio [AOR], 2.0; 95% confidence interval [CI], 1.5–2.7), high cholesterol (AOR, 2.2; 95% CI, 1.5–3.1), diabetes (AOR, 1.7; 95% CI, 1.2–2.4), and asthma (AOR, 2.2; 95% CI, 1.5–3.1) (Table 2). The association between smoking and FMD became significant (AOR, 1.5; 95% CI, 1.1–2.0). After further adjustment for age, sex, marital status, education, income, and employment, the association remained significant for hypertension, high cholesterol, diabetes, asthma, and smoking (Table 2). In the unadjusted (OR, 0.5; 95% CI, 0.4–0.8) and adjusted models (AOR, 0.6; 95% CI, 0.4–0.9), people reporting vigorous activity were significantly less likely to have FMD than those who did not report vigorous activity.

**Table 2:** The Prevalence and Odds of Frequent Mental Distress^a^ by Selected Chronic Conditions and Health Behaviors Among Adults Aged 18 Years or Older, Behavioral Risk Factor Surveillance Survey (BRFSS), Jordan, 2007

Conditions and Behaviors (Sample Size)	Frequent Mental Distress, % (95% CI)	OR (95% CI)	Age and Sex AOR (95% CI)	Multivariable^b^ AOR (95% CI)^c^
Hypertension (n = 3,553)				
Yes	17.3 (14.5–20.1)	2.2 (1.7–2.7)^d^	2.0 (1.5–2.7)^d^	2.0 (1.6–2.7)^d^
No	8.8 (7.7–9.8)	Reference	Reference	Reference
High cholesterol (n = 3,344)				
Yes	19.8 (15.1–24.6)	2.4 (1.7–3.3)^d^	2.2 (1.5–3.1)^d^	2.3 (1.6–3.2)^d^
No	9.4 (8.4–10.4)	Reference	Reference	Reference
Diabetes (n = 3,463)				
Yes	17.2 (12.7–21.7)	1.9 (1.4–2.7)^d^	1.7 (1.2–2.4)^d^	1.6 (1.1–2.4)^d^
No	9.6 (8.6–10.7)	Reference	Reference	Reference
Asthma (n = 3,607)				
Yes	20.2 (14.7–25.6)	2.4 (1.7–3.4)^d^	2.2 (1.5–3.1)^d^	2.2 (1.5–3.1)^d^
No	9.6 (8.6–10.6)	Reference	Reference	Reference
Current smoking (n = 3,612)				
Yes	11.1 (9.0–13.2)	1.1 (0.9–1.4)	1.5 (1.1–2.0)^d^	1.5 (1.1–2.0)^d^
No	10.0 (8.9–11.1)	Reference	Reference	Reference
Vigorous activity (n = 3,611)				
Yes	6.2 (4.2–8.3)	0.5 (0.4–0.8)^d^	0.6 (0.4–0.9)^d^	0.6 (0.4–0.9)^d^
No	10.9 (9.8–11.9)	Reference	Reference	Reference

Abbreviations: CI, confidence interval; OR, odds ratio; AOR, adjusted odds ratio.

a Defined as 14 or more mentally unhealthy days in the past 30 days.

b Multivariable includes adjusting for age, sex, marital status, education, income, and employment.

c Estimates and confidence intervals take into account the complex survey design of the Jordan BRFSS.

d Significant at *P* < .05.

## Discussion

In this study, we found FMD to be significantly associated with several chronic conditions and adverse health behaviors. Findings from several studies in the United States have also shown a potential link between chronic diseases, adverse health behaviors, and FMD (26–29). Our findings suggest that vigorous activity is protective against FMD, similar to findings in other published studies (27,28). The findings that FMD varied by age, sex, and income have also been found in the United States (30). In the United States, FMD was higher among adults aged 18–24 years (30). However, in Jordan, younger adults had the lowest prevalence of FMD compared with adults aged 35 years or older. In both the Jordan BRFSS and the United States, women were more likely to experience FMD than men as were people with a low income compared with those with a moderate or high income. Among people with FMD in Jordan, no differences were noted by marital status or education. However, in the United States, it has been noted that FMD is higher among separated people and people with less than a high school education (30). For this analysis, people who indicated they were separated were considered not married. As a result, our categorization of marital status into married and not married may have masked differences in FMD status. However, when examining sample size, the number of people reporting they were separated was less than 10.

This study has at least 4 limitations. The study was cross-sectional; thus, the temporal relationship between FMD and the exposures examined cannot be established. The information was self-reported and there may be recall bias associated with some of the responses. In addition, the health-related quality of life measure, mentally unhealthy days, although pilot tested in Jordan, was not validated in this population. We were unable to quantify vigorous activity because most responses were missing. As a result, the association observed between FMD and vigorous physical activity limits our interpretation because we could not incorporate frequency (days and minutes).

Since 2004, Jordan has had high prevalences of cardiovascular disease risk factors (smoking, lack of physical activity) and increasing numbers of people affected by diabetes, hypertension, and FMD. Our study provides insight into which groups (ie, Jordanians 50–64 years, people with low income, and people with chronic conditions such as hypertension and asthma) may be more likely to have FMD, a proxy for mental illness. The study results reflect a need for a concerted effort by the Jordan Ministry of Health to strengthen mental health care services, chronic disease prevention, and health promotion because both chronic diseases and mental health care services are offered in primary care. The 2010 report on mental health services in Jordan (22) and 2011 WHO Mental Health Atlas (21) both report that the Jordanian government has drafted a mental health policy and indicate that it should be approved in the near future. This policy is the first substantial step the Jordanian government has taken to manage mental health care in the country, and the government is to be commended for developing the policy. Suggested recommendations include 1) additional research to examine the complex relationship between FMD and chronic conditions; 2) using study findings to add to the limited information on mental health status in Jordan; 3) continued monitoring of FMD; and 4) training primary health care physicians in mental illness screening and treatment.
